# Disparities in Snacking Trends in US Adults over a 35 Year Period from 1977 to 2012

**DOI:** 10.3390/nu9080809

**Published:** 2017-07-27

**Authors:** Elizabeth K. Dunford, Barry M. Popkin

**Affiliations:** 1 Food Policy Division, The George Institute for Global Health, University of New South Wales, Sydney, NSW 2042, Australia; 2 Carolina Population Center, The University of North Carolina at Chapel Hill, Chapel Hill, NC 27516, USA; popkin@unc.edu; 3 Department of Nutrition, The University of North Carolina at Chapel Hill, Chapel Hill, NC 27516, USA

**Keywords:** energy intake, snacking, race–ethnic disparities, sugar-sweetened beverages

## Abstract

Background: Previous studies have indicated that snacking is contributing to increased calories in the American diet, and that the contribution of snacks to energy intake has increased in recent decades. Objective: The objective was to examine trends in the energy intake deriving from snacks and food sources of snacks for US adults over 35 years from 1977 to 2012, and whether these trends differ across sociodemographic groups. Methods: Participants included 74,291 US adults aged ≥19 years who participated in the 1977–1978 Nationwide Food Consumption Survey (NFCS); the 1989–1991 Continuing Survey of Food Intake by Individuals (CSFII), the 1994–1996 CSFII, the 1997–1998 CSFII, National Health and Nutrition Examination Survey (NHANES) 2003–2004, NHANES 2005–2006, NHANES 2009–2010 and NHANES 2011–2012; all surveys are a stratified random sample of the total civilian, non-institutionalized US population. Overall patterns of snacking, trends in energy intake from snacking, trends in snacking food and beverage sources and energy intake from snacks across racial/ethnic, age, education and income groups were examined. Results: For all US adults there was a significant increase in per capita energy intake from snacks from 1977 to 2012 (*p* < 0.01). Non-Hispanic blacks had the highest snacking energy intake from sugar-sweetened beverages (SSBs) in each year (*p* < 0.01 for all), with a peak intake in 2003–2006 of 76 kcal per capita per day. Intake of salty snacks increased in all groups between 1977 and 2012, with non-Hispanic blacks having the highest intake in 2009–2012 (*p* < 0.01). No significant differences were found between income or education groups. Conclusions: Results show that snacking remains a significant component of the US diet and the foods consumed at these snacks (SSBs, desserts and sweets and salty snacks) are not the types of foods recommended by the US dietary guidelines. Our finding that non-Hispanic blacks are consuming a higher proportion of SSBs and salty snacks than other groups, and showing the largest increase in energy intake deriving from snacks overall over the past 35 years, is indeed concerning.

## 1. Introduction

Large disparities have been documented in obesity and other chronic diseases across racial/ethnic and socio-economic groups in the US [1,2,3,4]. Meal and snack patterns can heavily influence health outcomes, particularly non-communicable diseases such as cardiovascular disease, Type II diabetes and obesity [5,6,7]. There are many dietary factors involved in the contribution to excess energy intake and obesity in adults including consuming food outside the home, increased portion sizes, increases in intake of sugar-sweetened beverages (SSBs) and increases in snacking behaviors [8,9,10,11].

In the US, snacks have been shown to comprise approximately 25% of daily energy intake, with a large proportion coming from salty snacks, desserts and sweets [12,13]. This is in spite of dietary guidelines recommending consumption of sweet, savory or salty snacks be limited [14]. An analysis of NHANES data from 1988 to 1994 found that Americans who reported consuming snacks as well as skipping a meal had less healthy overall intakes than people who ate three main meals [15]. However, there has been a limited number of evaluations of snacking trends over the past 10 years to evaluate whether snacking is still increasing in the US. One recent study looking at data from 1971 to 2010 reported an increase in snacking behavior over time for women [16]. With the most recent research indicating a decline in energy intakes and plateauing obesity levels over the past decade [17], it is important to examine whether similar changes in snacking behaviour are also occurring.

Previous research has highlighted important disparities in dietary intake and adherence to dietary guidelines between various racial/ethnic, education and income groups [18,19]. Higher income groups have been shown to have greater adherence to dietary intake recommendations, and non-Hispanic blacks have been shown to have the lowest adherence to the dietary guidelines [18]. However, to date, there has been limited published research examining differences in snacking behaviors in US adults by racial/ethnic group, household education or income level. One previous study looked at the number of snacking events across these various socio-demographic variables, showing that a higher proportion of non-Hispanic whites consume ≥6 snacks per day compared to other racial/ethnic groups [12]. Population averages may mask important differences between various socio-demographic subgroups, with evidence suggesting that certain subgroups have a higher prevalence of obesity and other risk factors for chronic disease [1,2,3,4].

As such, the objective of this paper was to examine changes in snacking patterns in US adults overall, by age group, racial/ethnic group, education and income level. Trends in energy intake from snacking, trends in snacking food and beverage sources, in addition to the trends of energy intake from snacks across various racial/ethnic, age, education and income groups were examined.

## 2. Materials and Methods

### 2.1. Survey Population

Data were obtained from eight nationally representative cross-sectional surveys of food intake in US adults between 1977 and 2012. The United States Department of Agriculture (USDA) data come from the 1977–1978 Nationwide Food Consumption Survey (NFCS); the 1989–1991 Continuing Survey of Food Intake by Individuals (CSFII), the 1994–1996 CSFII and the 1997–1998 CSFII. From the NHANES, 4 surveys were used: NHANES 2003–2004, NHANES 2005–2006 (NHANES 2003–2006), NHANES 2009–2010 and NHANES 2011–2012 (NHANES 2009–2012). Supplementary Table S1 shows the number of individuals with dietary data used in each survey year. The USDA and NHANES surveys are based on a multistage, stratified area probability sample of non-institutionalized U.S. households. Detailed information about each survey and its sampling design has been published previously [20,21,22,23,24]. In brief, the NFCS 1977–1978 and CSFII 1989–1991 surveys collected 3 consecutive days of dietary intake: one in-home, interviewer-administered 24-h recall and two self-administered 1-day food intake records. The CSFII 1994–1996 (CSFII 96) survey collected interviewer-administered 24-h recalls on 2 non-consecutive days (3–10 days apart). NHANES 2003–2012 collected two non-consecutive 24-h recalls, with the first day of intake by trained dietary interviewers and a second day by telephone interview. These interviews used a fully automated, computer-assisted multiple-pass dietary recall method. By utilizing secondary USDA and NHANES data, these analyses were exempt from institutional review board concerns.

### 2.2. Snacking Definition

Each eating occasion was self-defined by the respondent in each survey. Respondents were asked to name the type of each eating occasion [20,21,22,23,24]. The snack category includes foods and beverages consumed during those eating occasions defined by the respondent as “snack”, plus the occasions related to snacking, such as food and/or coffee/beverage breaks. The following responses were classified as a snack: ‘snack’, ‘botana’, ‘bebida’, ‘bocadillo’ and ‘tentempie’.

### 2.3. Dietary Data

To examine trends over time from surveys with different collection methods on days 1 and 2, only the first day’s data (a single, interviewer-administered 24-h dietary recall) collected from each individual (as recommended by the USDA) was used and used appropriate weights and adjustments for the sample design provided [25].

### 2.4. Food Grouping System

To determine those food items contributing to energy intake, the food grouping system developed by the University of North Carolina at Chapel Hill (UNC-CH) was used. This food grouping system links all foods from 1977 to the present to allow us to create consistent food groups. All the foods reported in the each survey were assigned to the 107 UNC-CH food groups [26]. For the purposes of this study, grain-based desserts and dairy desserts were combined to create a single “desserts” category, fruit and vegetable juices were combined into a single “juice” category to be consistent with previous papers using the UNC-CH food grouping system evaluating snacking behavior. For all individuals, the amount of energy provided by each UNC-CH snack food group was calculated and then divided by the total energy from snacking of all individuals. Those food groups contributing the most to snacking energy intake are reported.

### 2.5. Statistical Analysis

Data are presented as means. Snacking trends were studied by dividing the population into four age groups (19–29 years old, 30–59 years old, 19–59 years old and 60 years old and older), four racial/ethnic groups (Hispanic, Non-Hispanic White, Non-Hispanic Black and Other race), two education groups (Less than high school and High school graduate and higher) and three income groups (<185% National Poverty Level, 185–350% National Poverty Level and >350% National Poverty Level). Income definitions used self-reported family income to compute the family poverty level index. Survey commands were used to account for survey design, weighting, and clustering as per the NHANES analytic and reporting guidelines to researchers [25]. The proportion of adults consuming snacks within each racial/ethnic, income and education group was determined, as well as the proportion of snackers within each age group. The mean number of snacking occasions per day, mean energy intake (kcal) from snacks per capita per day, and the per capita intake deriving from snack foods within snackers only were calculated. The energy intake deriving from each food category by socio-demographic subgroups was also calculated. Data were analyzed using *t* test or one-way analysis of variance followed by the Bonferroni post hoc test and *p* ≤ 0.01 was considered statistically significant. The Bonferroni post hoc test correction was utilized to adjust for multiple comparisons. When examining differences in food group sources, differences between the 1977–1978 survey cycle and 2003–2006 survey cycle were compared to all other survey cycles. STATA version 14.1 (StataCorp, College Station, TX, USA) was used for all analyses [27].

## 3. Results

### 3.1. Snacking Trends by Age Group

For the overall US adult population there was a significant increase in per capita energy intake coming from snacks from 1977 to 2012 (*p* < 0.01) (Table 1) and the proportion of energy intake deriving from snack food sources. In 2003–2006 a peak in per capita energy intake from snacks was seen in most age groups, with a subsequent decrease between 2006 and 2012, however this result was only significant in the 19–29 year age group (*p* < 0.01) (Table 1). In ages 30–59 years the same trend of a steady increase between 1977 and 2006 and then a subsequent decrease between 2006 and 2012 was seen in the number of snacks per day, the percentage of snackers in the population and the energy content per snack. However, in US adults aged 60+ years the energy content per snack increased over the study period from 1977 to 2012 and did not show a decrease between 2006 and 2012.

### 3.2. Overall Food and Beverage Shifts

**Figure 1:**
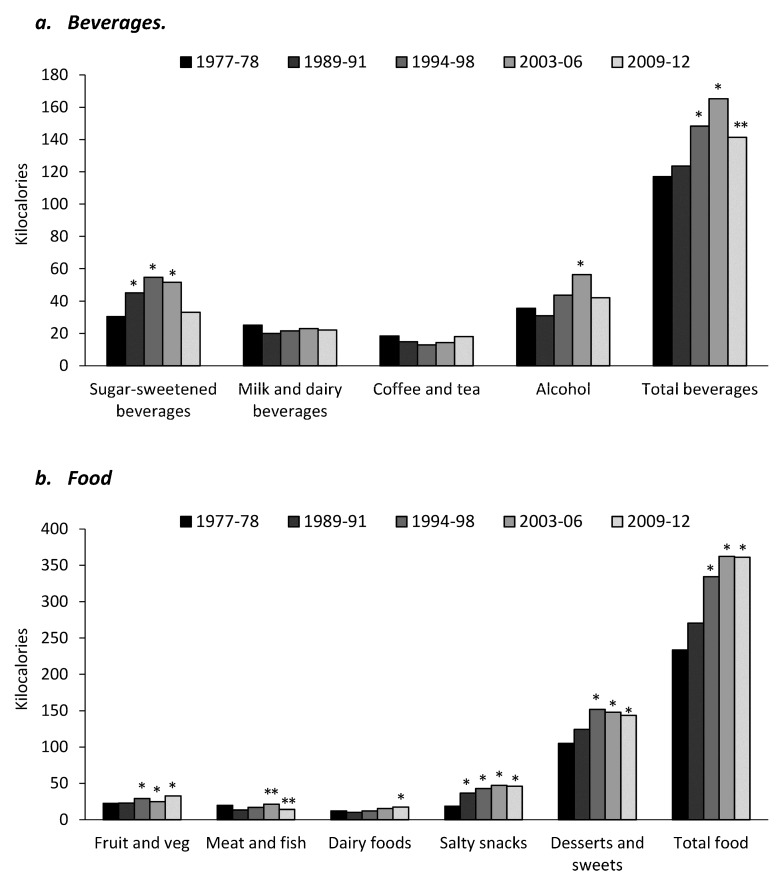
Per capita intake (kcal) from snacks—(**a**) beverages and (**b**) food sources for US adults. * Significantly different to 1977–1978, *p* < 0.01; ** Significantly different to 2003–2006, *p* < 0.01.

Per capita intake from snacks from beverage and food sources is shown in Figure 1. For both food and beverage intake the trend was an increase in intake of salty snacks and desserts and sweets from 1977 to 2012 (Figure 1b) and SSBs and alcohol showing an increase from 1977 to 2006, and then a large decline from 2006 to 2012 (Figure 1a). Milk and dairy beverage intake and coffee and tea intake remained stable over the study period. Intake of dairy foods and fruit and vegetables was significantly higher in 2009–2012 than in 1977–1978 (*p* < 0.01), however intake of meat and fish significantly declined (Figure 1b).

### 3.3. Snacking Trends by Racial/Ethnic Group

**Figure 2:**
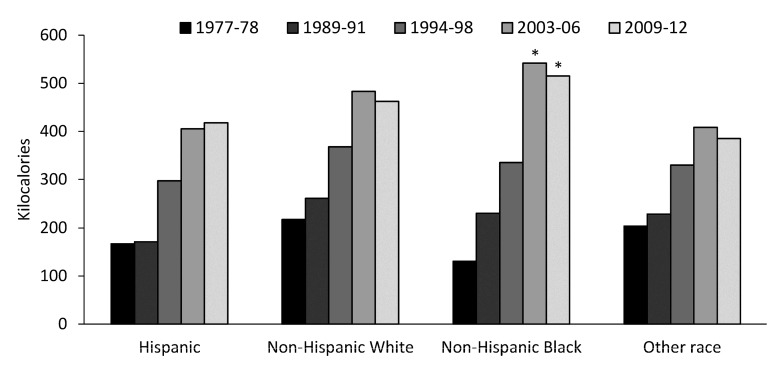
Kilocalories per capita per day from snacks by ethnic group for US adults. * Significantly different to all racial-ethnic groups, *p* < 0.01.

**Figure 3:**
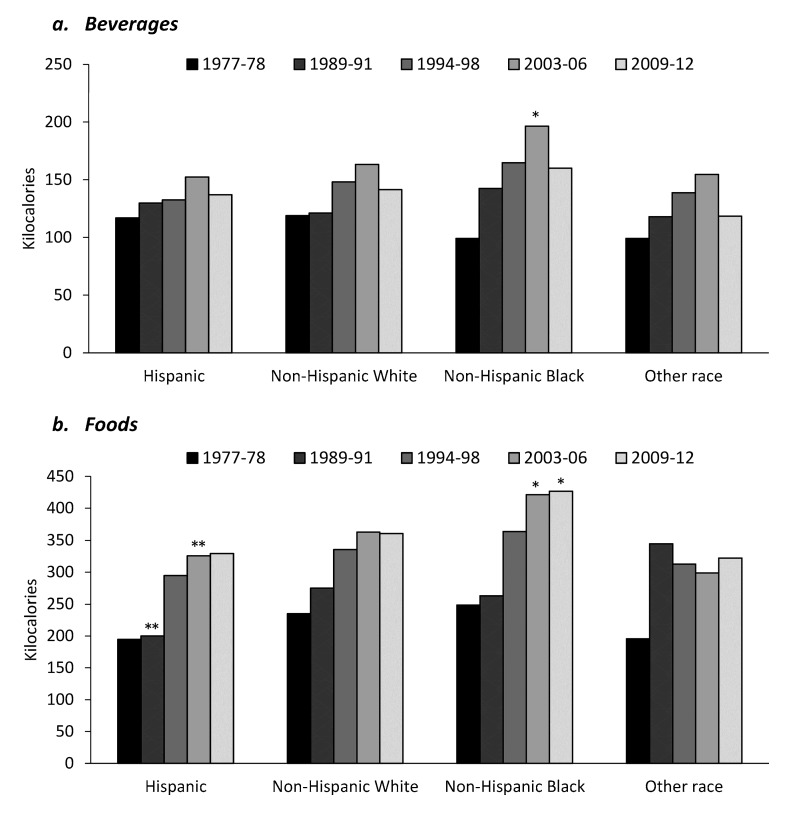
Kilocalories per capita per day from (**a**) beverages and (**b**) foods by ethnicity for US adults. * Significantly different to all racial-ethnic groups, *p* < 0.01. ** Significantly different to non-Hispanic white, *p* < 0.01.

All racial/ethnic groups showed an increase in kcal per capita per day from snacks between 1977 and 2006 (*p* < 0.01 for all) and then a decline in kcal per capita per day from snacks between 2006 and 2012 except for Hispanics which showed a continued increase from 2006 to 2012 (Figure 2). Across all racial/ethnic groups there was a slight increase in energy intake deriving from beverage snack sources from 1977 to 2012 (Figure 3a). Energy intake for food snack sources increased by more than 50% in all racial/ethnic groups from 1977 to 2012 (*p* < 0.01 for all), with non-Hispanic blacks showing the largest increase both in food snack sources (Figure 3b) and in kcal from snacks overall (Figure 2). In 1977–1978, non-Hispanic blacks had the lowest energy intake deriving from snacks (131 kcal per capita per day) but the highest intake in 2009–2012 (515 kcal per capita per day; *p* < 0.01) (Table 1). When results were restricted to look at only those who consumed snacks, non-Hispanic blacks on average consumed 102 kcal per capita per day more than the next highest racial/ethnic group (Table 1).

### 3.4. The Major Food and Beverage Drivers of the Trends

Overall, per capita kcal intake from SSBs as a snack increased from 1977–1978 to 1994–1998 and then steadily decreased from 1998 to 2009–2012 (Supplemental Figure S1a). Non-Hispanic blacks had the highest calorie intake from SSBs in each survey year (*p* < 0.01 for all except 1977–1978), with a peak of intake in 2003–2006 of 76 kcal per capita per day. Non-Hispanic blacks also had the highest intake of salty snacks out of all racial/ethnic groups in both 2003–2006 and 2009–2012 (*p* < 0.01 for all) (Supplemental Figure S1b). Intake of desserts and sweets increased in all racial/ethnic groups between 1977 and 1994–1998 and then declined slightly, with non-Hispanic blacks having the highest intake in 2009–2012, with more than 160 kcal per capita per day deriving from desserts and sweets (Supplemental Figure S1c) compared to, for example, “other” race with 106 kcal per capita per day.

In all racial/ethnic groups, grain-based desserts were the number one source of total kcal intake in 2009–2012 (Supplementary Table S2), with 60% of the top food sources of snacks deriving from food items such as salty snacks, desserts and sweets and SSBs for all racial/ethnic groups. Non-Hispanic blacks were the only group to not have fluid milk as a top 10 contributor to intake of energy from snacks.

## 4. Discussion

This is the first study to examine adult snacking trends during the past 10 years in the US population by racial/ethnic group. Results showed that in all sociodemographic groups there was a significant increase in the per capita energy intake coming from snacks from 1977 to 2012 (*p* < 0.01). Overall calories from snacking among adults, particularly those 19–29 years, remains high at about 500 kcal per day with the bulk coming from foods generally considered unhealthy such as desserts, SSBs and salty snacks.

Although there is no published research on racial/ethnic disparity snacking trends in US adults to date, this study’s results show that non-Hispanic blacks are consuming a higher intake of kcal from snacks than other groups are consistent with recent US research in children and adolescents looking at behavioral shifts in food purchases which found that non-Hispanic blacks were the only racial/ethnic group not to follow the overall trend of a decrease in per capita energy intake in the last decade [16], as well as research showing that non-Hispanic blacks overall have less healthy food purchasing behavior than white and Hispanic populations [28]. Other research, however, has shown that non-Hispanic whites are more likely to consume a higher number of snacks compared to other racial/ethnic groups [12], however these results were found using information from two 24 h recalls rather than from one as used in the present analysis. Prior studies in smaller samples of the US population have supported our results, showing less healthy snacking behaviors (in particular, higher intake of SSBs) in non-Hispanic black adolescents [29,30,31] than in other groups. This, along with the high intake of energy from SSBs, salty snacks and desserts and sweets observed in non-Hispanic blacks in this study is concerning, with obesity prevalence and its risk factors shown to be considerably higher among non-Hispanic blacks compared to whites [32].

Overall, the majority of energy intake from snacks for US adults were from sources generally considered less healthy such as desserts and sweets, salty snacks, alcohol and SSBs. This fits with research showing that the US population overall has a high intake of foods high in added sugar and solid fats [33]. It also supports research from other western countries that the majority of energy intake from snacks derives from less healthy foods [34,35]. Interestingly, although the per capita intake of these “less healthy” food sources has increased more than 50% from 1977 to 2012, the proportion of total snacking intake deriving from these sources did not show the same trend, and has remained unchanged from 1977 to 2012. This is consistent with previous research examining snacking food sources in US adults which has shown that a large percentage derives from unhealthy food sources such as salty snacks, desserts and sweets and SSBs [12]. In fact, our 2012 data showed that for all racial/ethnic groups, desserts and sweets were the number one source of energy from snacking. Another important result to note is that alcohol intake increased from 1977–1978 to 2003–2006 and declined from 2003–2006 to 2009–2012. Excessive alcohol intake has been linked to negative health outcomes, and is also not recommended in the dietary guidelines.

This analysis had some limitations. Similar to other studies looking at US trends in dietary intake, different methodologies were used, particularly the shift from the 1970s to the 1990s. The introduction of the multiple pass method in 1994 may have resulted in additional snacks during that period; however, the finding of a recent decrease in snacking overall provides some reassurance that this did not affect our overall findings. Earlier survey years conducted without the multiple pass method may be more prone to issues of recall bias. There is no bridging survey to help understand the impact of this methodological change, meaning that these surveys are the only ones available that use consistent food composition tables developed by the USDA specifically for the food supply at the time of each survey. Each survey was linked to Food and Nutrient Database for Dietary Studies (FNDDS) USDA food composition tables but there may have been changes in nutrient composition based on different assay techniques for which we cannot account [36,37]. These concerns were addressed by using the food grouping system developed by UNC which allows foods in each survey year to be linked to one consistent food group to offset changes in food composition table numbering and ensure high quality estimates of nutrient values over time [38]. It is also a possibility that the Spanish terms in NHANES used to describe a ‘snack’ may have multiple meanings, and results should be interpreted with the understanding that snacking in the Hispanic population may have been overestimated. Similarly, how Americans define a ‘snack’ may have changed over time, and should also be considered when interpreting the results. However, our finding that in the most recent survey cycles that snacking has decreased gives us faith that the results shown are representative of true snacking trends that have occurred since 1977–1978.

## 5. Conclusions

This study shows important changes in the number of snacking occasions, foods consumed, and total contribution of snacks to overall energy intake across various racial/ethnic groups of US adults. Importantly, it also shows that snacking behavior in the US is different between different racial/ethnic groups, and that snacking remains a significant component of the US diet and the foods consumed at these snacks (SSBs, alcohol, desserts and sweets and salty snacks). These foods are not the types of foods recommended by the US dietary guidelines [14]. These findings have implications for the foods and behaviors that future interventions or policy might target to improve racial/ethnic disparities in the US population related to diet. Further studies should examine how these increases in less healthy snacking behaviors among vulnerable populations may contribute to disparities in diet and health. It will also be important to examine the results of future dietary surveys to determine whether the recent decrease in calorie intake from snacking continues.

## Figures and Tables

**Table 1:** Snacking behaviors of US adults from 1977 to 2012 by age and racial/ethnic group.

	NFCS ^a^ 1977–1978	CSFII ^b^ 1989–1991	CSFII ^b^ 1994–1998	NHANES ^c^ 2003–2006	NHANES ^c^ 2009–2012
**Age 19–29 years**					
Snacks, *n*/day	1.1 ^d^	1.1	1.5	2.2	2.2
Per capita mean intake from snacks, kcal/day	262 ^d^	300 ^d^	470	582 ^d^	516
% snackers	60 ^d^	61	73	86	85
Per capita mean intake from snacks, kcal/day (snackers only)	439 ^d^	489 ^d^	643	675	607
**Age 30–59 years**					
Snacks, *n*/day	1.1 ^d^	1.3	1.6	2.4	2.3
Per capita mean intake from snacks, kcal/day	208 ^d^	258 ^d^	360 ^d^	497	484
% snackers	61 ^d^	65	74	88	87
Per capita mean intake from snacks, kcal/day (snackers only)	344 ^d^	396 ^d^	483 ^d^	566	554
**Age 60+ years**					
Snacks, *n*/day	0.8 ^d^	1.1	1.3	2.1	2.1
Per capita mean intake from snacks, kcal/day	135 ^d^	181 ^d^	241 ^d^	342	347
% snackers	50 ^d^	60	71	87	85
Per capita mean intake from snacks, kcal/day (snackers only)	270 ^d^	301 ^d^	342 ^d^	395	407
**Age 19–59 years**					
Snacks, *n*/day	1.1 ^d^	1.3	1.6	2.3	2.3
Per capita mean intake from snacks, kcal/day	226 ^d^	271 ^d^	390 ^d^	520	493
% snackers	60 ^d^	64	74	87	87
Per capita mean intake from snacks, kcal/day (snackers only)	375 ^d^	422 ^d^	527 ^d^	595	569
**Hispanic**					
Snacks, *n*/day	0.9 ^d^	0.9	1.2	2.0	2.1
Per capita mean intake from snacks, kcal/day	167 ^d^	171 ^d^	297 ^d^	405	418
% snackers	53 ^d^	52	69	82	84
Per capita mean intake from snacks, kcal/day (snackers only)	312 ^d^	330 ^d^	429 ^d^	495	495
**Non-Hispanic White**					
Snacks, *n*/day	1.1 ^d^	1.3	1.6	2.4	2.3
Per capita mean intake from snacks, kcal/day	217 ^d^	261 ^d^	368 ^d^	483	462
% snackers	61 ^d^	66	75	89	88
Per capita mean intake from snacks, kcal/day (snackers only)	357 ^d^	398 ^d^	488	545	527
**Non-Hispanic Black**					
Snacks, *n*/day	0.6 ^d^	1.0	1.1	2.0	2.1
Per capita mean intake from snacks, kcal/day	131 ^d^	230 ^d^	335 ^d^	542	515
% snackers	38 ^d^	57	62	84	83
Per capita mean intake from snacks, kcal/day (snackers only)	346 ^d^	406 ^d^	544	643	629
**Other Race**					
Snacks, *n*/day	1.1 ^d^	0.9	1.7	2.1	2.2
Per capita mean intake from snacks, kcal/day	204 ^d^	228 ^d^	330	408	385
% snackers	68 ^d^	49	73	85	84
Per capita mean intake from snacks, kcal/day (snackers only)	299 ^d^	462	451	481	460

^a^ NFCS = Nationwide Food Consumption Survey; ^b^ CSFII = Continuing Survey of Food Intakes by Individuals; ^c^ NHANES = National Health and Nutrition Examination Survey; ^d^ Different from 2009 to 2012; *p* < 0.01.

## Supplementary Materials

The following are available online at www.mdpi.com/2072-6643/9/8/809/s1, Figure S1: Per capita daily energy intake (kcal) for snacks by race/ethnicity for US adults between 1977–1978 and 2009–2012, Table S1: Sociodemographic characteristics for US adults ages ≥19 years from the 1977–1978 Nationwide Food Consumption Survey (NFCS); the 1989–1991 Continuing Survey of Food Intake by Individuals (CSFII), the 1994–1996 CSFII, the 1997–1998 CSFII, NHANES 2003–2004, NHANES 2005–2006, NHANES 2009–2010 and NHANES 2011–2012, Table S2: Ranking of food and beverage sources of snacks by ethnic group (2009–2012).
