# Supplementary material for: Disparities in Snacking Trends in US Adults over a 35 Year Period from 1977 to 2012
Source: Nutrients. 2017 Jul 27;9(8):809. doi: 10.3390/nu9080809 (PMC5579603; doi:10.3390/nu9080809)

Supplementary Table S1. Sociodemographic characteristics for US adults ages ≥19 years from the 1977–1978 Nationwide Food Consumption Survey (NFCS); the 1989–1991 Continuing Survey of Food Intake by Individuals (CSFII), the 1994–1996 CSFII, the 1997-1998 CSFII, NHANES 2003–2004, NHANES 2005– 2006, NHANES 2009-2010 and NHANES 2011-2012.

	**NFCS 1977-78**	**CSFII 1989-91**	**CSFII 1994-98**	**NHANES 2003-06**	**NHANES 2009-12**
Number of observations	29,715	11,877	10,019	10,577	12,103
Female (%)	53.6	56.3	48.8	51.9	51.0
Male (%)	46.4	43.7	51.2	48.1	49.0
Hispanic (%)	5.6	8.9	8.9	23.5	24.7
Non-Hispanic white (%)	80.9	75.5	79.3	39.4	42.2
Non-Hispanic black (%)	12.4	12.6	11.2	26.3	22.2
Other race (%)	1.1	3.0	0.6	4.8	11.0
<185% national poverty level (%)	29.2	51.9	35.0	39.6	44.6
185-350 % national poverty level (%)	36.7	23.7	27.9	24.8	19.7
>350% national poverty level (%)	34.1	24.4	37.1	29.8	26.1
Household's education <= high school diploma (%)	24.1	30.4	21.0	29.0	26.4
Household’s education >= high school diploma (%)	75.7	69.6	77.4	70.8	73.5

Supplementary Table S2. Ranking of food and beverage sources of snacks by ethnic group (2009–2012).

**Snack food source**	**Hispanic**	**NH White**	**NH Black**	**Other Race**
Desserts and sweets	1	1	1	1
Fruit, fresh, frozen, canned, or dried	2	6	6	2
Sugar sweetened beverages	3	4	3	5
Beer	4	5	4	6
Salty snacks	5	2	2	3
Corn and flour tortilla/corn-based dishes	6	9	-	-
Fluid milk	7	7	-	8
Nuts, nut butters, seeds, & coconut	8	3	5	4
Coffee/tea	9	8	8	7
Breads and rolls, mostly unsweetened	10	-	7	9
Cheese & cheese products	-	10	-	-
Liquor	-	-	9	-
Juices	-	-	10	10

Figure S1. Per capita daily energy intake (kcal) for snacks by race/ethnicity for US adults between 1977–1978 and 2009–2012. * Significantly different to all racial-ethnic groups, $p < 0.01$. ** Significantly different to non-Hispanic White, $p < 0.01$.

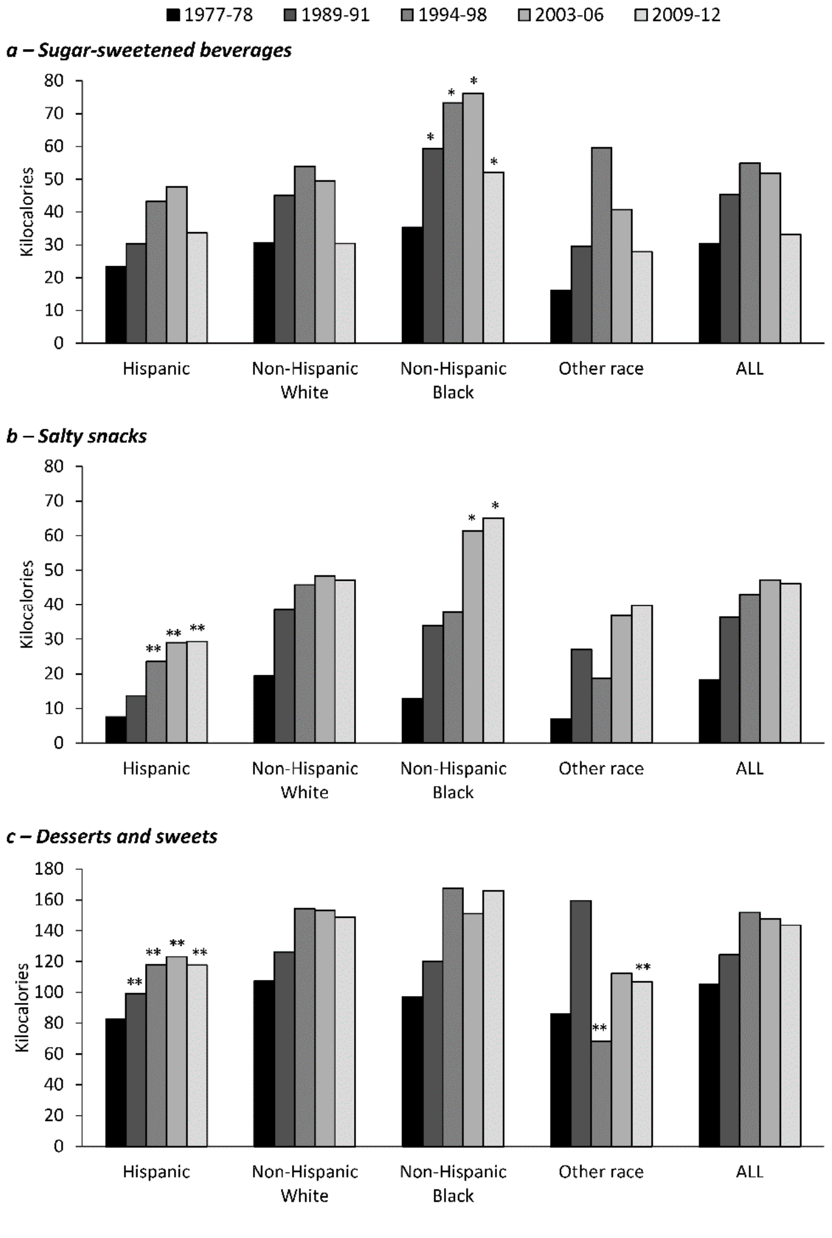

Supplement: Supplementary file 1 [file nutrients-09-00809-s001.pdf]
